# Acute antagonism in three-drug combinations for vaginal HIV prevention in humanized mice

**DOI:** 10.1038/s41598-023-31695-5

**Published:** 2023-03-21

**Authors:** Philippe A. Gallay, Christina M. Ramirez, Marc M. Baum

**Affiliations:** 1 Department of Immunology and Microbiology, The Scripps Research Institute, 10550 North Torrey Pines Road, La Jolla, CA USA; 2 Department of Biostatistics, UCLA Fielding School of Public Health, University of California, Los Angeles (UCLA), Los Angeles, CA USA; 3 Department of Chemistry, Oak Crest Institute of Science, 128-132 W. Chestnut Ave., Monrovia, CA USA

**Keywords:** HIV infections, Microbiology, Medical research, Pharmacology

## Abstract

Adolescent girls and young women in low- to middle-income countries are disproportionately at risk of becoming HIV-1 infected. New non-vaccine biomedical products aimed at overcoming this global health challenge need to provide a range of safe, effective, and discreet dosage forms based on the delivery of one or more antiviral compounds. An overarching strategy involves vaginal drug administration through inserts/tablets, gels, films, and intravaginal rings. The approach derives its appeal from being women-controlled and topical, there-by potentially minimizing systemic exposure to the agents and their metabolites. Oral regimens based on tenofovir disoproxil fumarate (TDF) and emtricitabine (FTC) are established and effective in HIV-1 pre-exposure prophylaxis (PrEP), and form a promising basis for vaginal PrEP. Here, we used bone marrow/liver/thymus humanized mice to measure the in vivo efficacy against HIV-1 of single and combination antiviral compounds applied vaginally, coupled with data analysis using the Chou-Talalay mathematical model to study the dose–effect characteristics. Unexpectedly, strong antagonism was observed in drug combinations composed of TDF-FTC coupled with a third agent using a different mode of action against HIV-1. The antagonistic effect was remedied when TDF was omitted from the regimen. Our approach provides a translational template for the preclinical, rational, and systematic evaluation of drug combinations for the prevention of HIV-1, and other viral diseases.

## Introduction

A concerning HIV-1 prevention gap persists despite aggressive, multifaceted global efforts aimed at eradicating the epidemic, with the number of annual, new HIV-1 infections stalling around 1.7 million in 2019^1^. Adolescent girls and young women (AGYW, ages 15–24) are disproportionately at risk, with an estimated 7,000 new HIV-1 infections occurring weekly^2^. In sub-Saharan Africa, three in four new infections are among girls aged 15–19 years^2^. Consequently, highly effective, non-vaccine biomedical modalities for HIV-1 prevention in AGYW are needed urgently.

An oral regimen of the antiretroviral (ARV) agents tenofovir disoproxil fumarate (TDF) and emtricitabine (FTC) is effective for HIV-1 pre-exposure prophylaxis (PrEP) and is being implemented in numerous countries^3^, but discontinuation is high in multiple populations^4–7^. New, complementary HIV-1 PrEP products developed for AGYW in low- to medium-income countries are needed to expand the palette of HIV-1 prevention options that meet safety and efficacy targets, along with being discreet, portable, and women-controlled in a manner that is appealing to the intended end-users. Vaginal drug delivery (e.g., gels, films, inserts/tablets, intravaginal rings) theoretically meets these requirements while often minimizing systemic exposure to the antiviral agents and their metabolites.

An intravaginal ring (IVR) delivering the non-nucleoside reverse transcriptase inhibitor dapivirine (DPV) was found to be safe and effective at preventing HIV-1 infection in two large Phase III clinical trials^8,9^. In March 2022, the monthly DPV IVR was granted regulatory approval by the South African Health Products Regulatory Authority (SAHPRA) for use by women ages 18, and older, to reduce their risk of HIV acquisition. The approval marked an important milestone by offering a much-needed, woman-controlled HIV-1 PrEP option. End-user preference studies involving AGYW in sub-Saharan Africa showed that a range of complementary product options, topical and systemic, likely will be needed to help bridge the HIV-1 prevention gap^10–13^. Consequently, a number of promising topical HIV-1 PrEP product candidates are advancing into early-stage clinical trials, including: fast-dissolving tenofovir vaginal film^14^; MB66, a multipurpose prevention vaginal film delivering the monoclonal antibodies (mAbs) against HIV-1 (VRC01-*N*) and HSV-1 and 2 (HSV8-*N*)^15^; vaginal fast-dissolving insert combining griffithsin and carrageenan^16^; single dose topical insert containing tenofovir alafenamide fumarate and elvitegravir^17^; IVRs delivering TDF, TDF-FTC, and TDF-FTC combined with maraviroc^18^; and 90-day tenofovir plus levonorgestrel IVR^19^. The vibrant field of topical HIV-1 PrEP is reliant on rigorous pharmacodynamic evaluation of the vaginally administered agents, alone or in combination.

The preclinical evaluation of vaginal anti-HIV-1 efficacy in suitable animal models forms an important basis in the rational and systematic selection of suitable antiviral prevention agents, either to be used alone or in combination. A vaginally applied drug regimen based on a TDF-FTC foundation could build on the established success of oral (i.e., systemic) PrEP, but it is not clear if a third agent using a different mode of action against HIV-1 would be beneficial. The road to realizing the potential of potent, vaginal HIV-1 prevention products requires the evaluation of drug combinations in a meaningful animal model that can distinguish between additive, synergistic, or antagonistic effects.

Herein, we describe vaginal HIV-1 prevention efficacy studies in in bone marrow/liver/thymus (BLT) humanized (hu) mice, an established model in HIV-1 research^20^, using topically applied single, dual, and triple antiviral drug combinations. The measured dose–effect characteristics were analyzed empirically using the Chou-Talalay mathematical model^21–23^, allowing rigorous evaluation of drug interactions based on the median-effect principle of the mass action law. Unexpectedly, strong antagonism was observed in three-drug combinations based on TDF-FTC that was remedied when TDF was omitted. The implications of our findings in terms of drug selection for development of potent vaginal HIV-1 PrEP regimens are discussed.

## Results

### BLT hu-mouse study design and efficacy endpoints

The BLT hu-mouse model is well-established in HIV-1 prevention research^20,24^. Humanization of the immune system in these mice is achieved by implanting human fetal liver and thymus tissues under the kidney capsule of immunodeficient NOD-scid gamma chain knockout (NSG), followed by administration of autologous human fetal liver CD34^+^ cells (human hematopoietic stem and progenitor cells, HSPCs). T-cell education occurs in the human thymic tissue leading to complete systemic reconstitution of all major human hematopoietic lineages, including T, B, monocyte/macrophage, dendritic, and natural killer cells. Importantly, the extensive systemic and genital mucosal reconstitution with human lymphoid cells renders female BLT hu-mice susceptible to vaginal HIV-1 infection. We verified the degree of humanization of the BLT mice at 20 weeks of age, 10 weeks post-CD34^+^ HSPC injection, as in previous studies^25–27^. Only mice that met the humanization targets determined previously^28^ (> 65% of CD45^+^ cells and > 70% of CD3^+^ and CD4^+^ cells) were used in infection studies.

**Figure 1:**
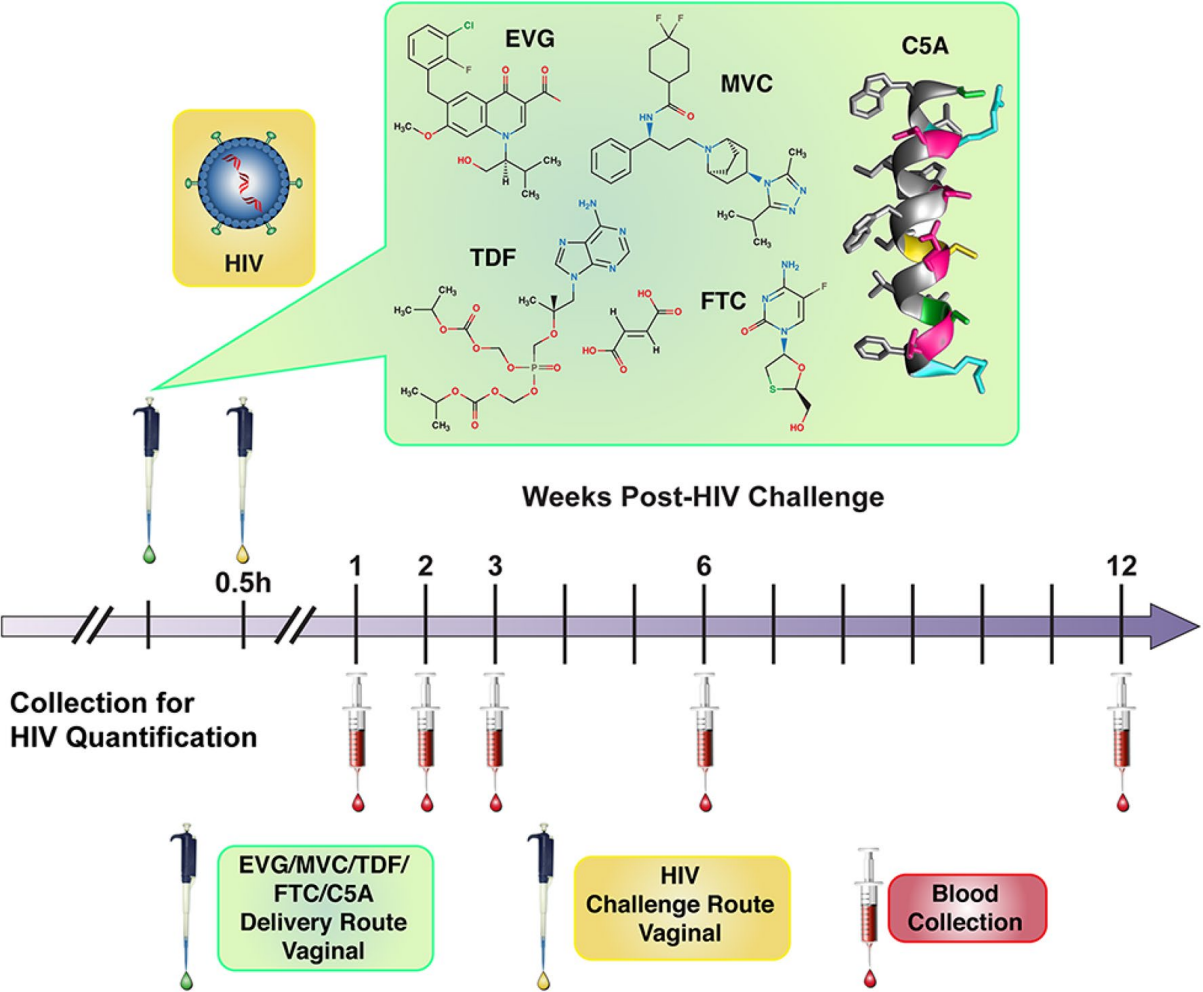
Experimental design of BLT hu-mouse vaginal HIV-1 prevention studies. Vaginal application of drug solution in phosphate-buffered saline (PBS, green box) was followed by the HIV-1 exposure (yellow box) within 30 min (typically 15–25 min). Peripheral blood samples were collected at the indicated times (red box) and HIV-1 viral load measured by qPCR.

The overall vaginal HIV-1 prevention study design using female BLT hu-mice has been described previously^25–27^, and is shown schematically in Fig. 1. Either 8 or 10 mice per group were employed. The statistical power resulting from these group sizes was calculated using two-sided binomial tests, and has been reported previously^27^. Viral RNA was measured in plasma at 1, 2, 3, 6, and 12 weeks post challenge to determine if infection occurred.

A diagonal, constant, equipotency ratio combination design^21^ (see Supplementary Data S1–S4 for drug dosing concentrations) was used to minimize the number of animal groups used, while still allowing the quantitative study endpoints to be met. Typically, 5–6 drug concentrations per group were used (*N* = 8–10 mice each, vide supra). Initially, we determined the ED_50_ values (the dose required to achieve 50% protection from HIV-1 infection) for drugs administered individually, allowing combination experiments to be designed. The ED_50_ and ED_90_ values calculated here allow for standardized comparison of drug potencies and form important inputs to the empirical analysis of drug combination effects (vide infra). However, they should not be viewed at concentration targets for vaginal HIV-1 PrEP products, as these would need to be higher, predicated by safety, to maximize duration of efficacy.

For three-drug combinations, we created equipotency ratio combinations based on multiples (1/3, 1, and 3) of the individual drugs’ vaginal ED_50_ values (Supplementary Data S3). A subsequent study using three additional dosing groups determined based on the pilot study results was used to fill in gaps in the dose–response relationships (Supplementary Data S4). This approach was facilitated by the high reproducibility in humanization of the mice observed in previous studies^25–27^.

### Dose–response and slope parameters for single antiretroviral drug regimens

We have previously measured the vaginal HIV-1 prevention dose–response characteristics of single agents spanning a range of antiviral mechanisms of action, namely: TDF^25^, a prodrug and nucleotide analog reverse transcriptase inhibitor competing with adenosine monophosphate; FTC^25^, a nucleoside analog reverse transcriptase inhibitor competing with cytidine; C5A^26^, a broadly antiviral, 18-amino acid, linear peptide that disrupts the viral membrane; and VRC01-*N*^27^, a broadly neutralizing antibody (bNAb) against HIV-1. Here, we add efficacy data for two further ARV drugs from different mechanistic classes to our in vivo HIV-1 prevention dataset.

**Figure 2:**
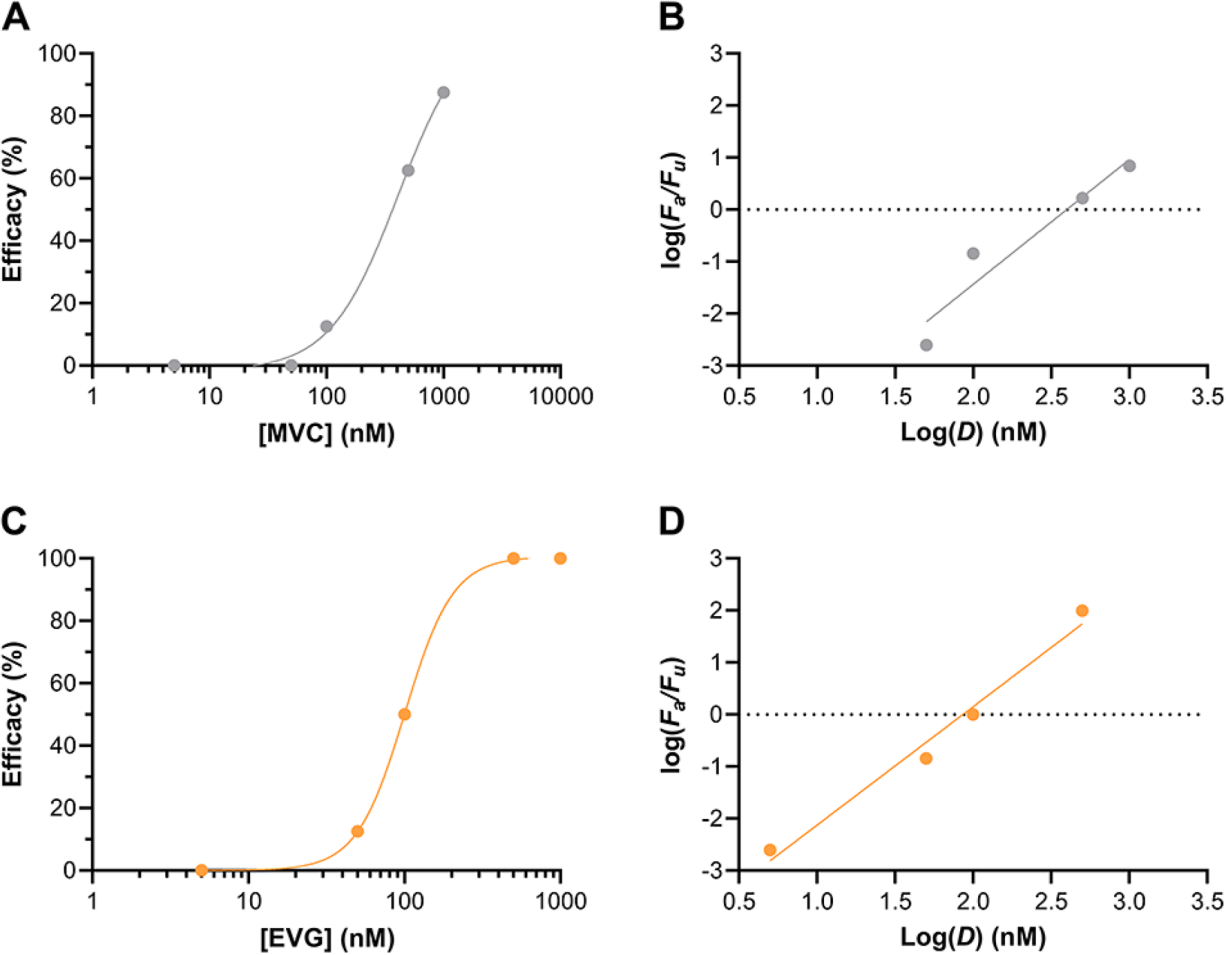
Dose–response curves for vaginal HIV-1 challenge studies in BLT hu-mice. Plots of efficacy *versus* dose of (**A**) MVC (*N* = 8 per dosing group, 5 groups) and (**C**) EVG (*N* = 8 per dosing group, 5 groups) applied prior to HIV-1 challenge, analyzed using a variable slope model (four-parameter dose–response curve) that does not constrain the Hill slope. Median-effect plots for the corresponding vaginal HIV-1 efficacy studies using (**B**) MVC and (**D**) EVG dosing represent a different model for data analysis. *F*_*a*_, fraction affected; *F*_*u*_, fraction unaffected; *D*, dose (nM).

The dose–response relationships for the chemokine co-receptor 5 (CCR5) antagonist maraviroc (MVC, *N* = 8 per dosing group, 5 groups, Supplementary Data S1) and the integrase strand transfer inhibitor (ISTI) elvitegravir (EVG, *N* = 8 per dosing group, 5 groups, Supplementary Data S2) are presented in Fig. 2.

The compounds exhibited sigmoidal dose–effect HIV-1 prevention curves analogous to those observed in our previous studies with TDF, FTC, C5A, and VRC01-*N*. Key pharmacodynamic (efficacy) parameters from the single drug systems shown in Fig. 2, along with data from previous studies^25,26^, re-analyzed using boundary conditions consistent with the current analysis (see “Methods”), were calculated using the median-effect Eq. ^21^ and are presented in Table 1.

**Table 1:** Dose–response characteristics of vaginally applied agents in BLT hu-mice derived from median-effect model analyses.

Anti-HIV-1 Drug	ED_50_ (µM)	ED_90_ (µM)	*m*-value	*R*^*2*^
TDF^a^	4.8	9.1	3.47	0.980
FTC^a^	0.16	0.35	2.79	0.962
C5A^b^	37	58	4.90	0.986
MVC^c^	0.40	0.99	2.40	0.958
EVG^c^	0.086	0.23	2.28	0.990

^a^See Gallay et al.^25^.

^b^See Gallay et al.^26^.

^c^This work.

The median-effects plots^21,22^ allowed a number of key parameters to be calculated (Fig. 2B,D, Table 1), based on the condition that the mass-action principle be followed by the experimental system under investigation. This criterion, defined by *R*^*2*^ values greater than 0.9 for in vivo studies^21,22^, was met in the above vaginal challenge studies (Table 1). The *x*-intercept [*y* = log (*F*_*a*_/*F*_*u*_) = 0] corresponds to the potency parameter, *D*_*m*_, or the dose needed to achieve the median-effect, denoted here as ED_50_. The slope, or *m*-value, is the shape parameter and defines the sigmoidicity of dose–effect curve. In cases when *m* = 1, the dose–effect curve is hyperbolic (e.g., Michaelis–Menten kinetics), while it is sigmoidal when m ≠ 1. The greater the *m*-value, the greater the sigmoidicity leading to steeper dose–response relationship (i.e., lower increase in dose concentration needed to achieve a given increase in effect, *F*_*a*_), a desirable characteristic for HIV-1 PrEP. Finally, *m* < 1 represents a relatively rare system, typically suggestive of allosterism via drug binding at receptor or enzyme sites (i.e., negative cooperativity).

We selected EVG and C5A as antiviral agents for further evaluation as triple-drug combinations with TDF-FTC (vide infra) based on the understanding that both agents have widely disparate mechanisms of action. The ISTI EVG acts in HIV-1 target cells supporting viral replication, while the antiviral peptide C5A disrupts the viral envelope before HIV-1 binds host cells^26,29^. We omitted MVC from additional studies in part based on funding limitations, and because it had *ca.* fivefold lower potency compared to EVG (ED_90_, Table 1). Council et al. used the BLT hu-mouse model to show that topically applied MVC prevented vaginal HIV-1 infection in the presence of semen^30^. Both EVG and MVC are FDA-approved ARV agents to treat HIV-1 infection that are being explored for vaginal HIV-1 prevention^18,31–33^ and we plan to include MVC in future studies.

### Empirical analysis of drug combination effects on HIV-1 preventative efficacy

**Figure 3:**
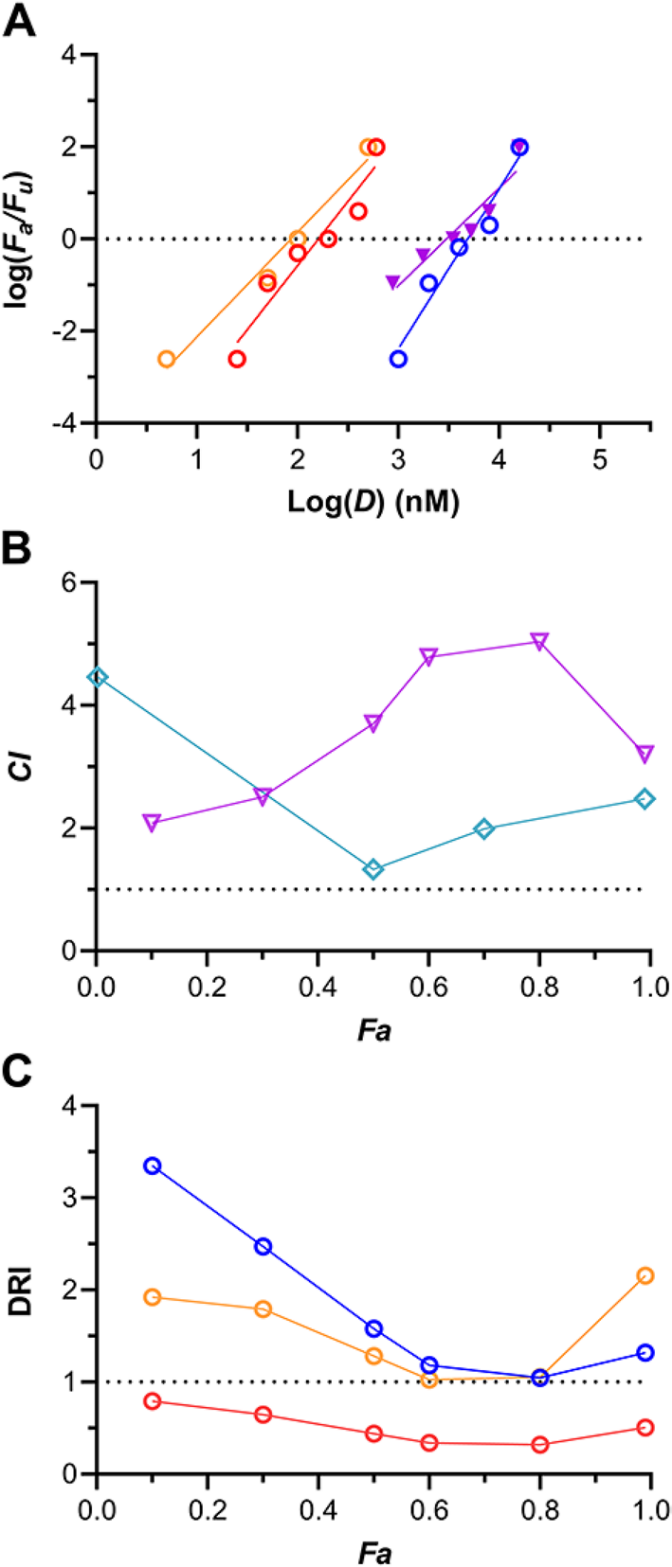
Median-effect model analysis of efficacy in vaginal HIV-1 prevention using BLT hu-mice (*N* = 10 per dosing group, 6 groups per study) and dosing with a triple drug combination consisting of TDF-FTC-EVG. *F*_*a*_, fraction affected; *F*_*u*_, fraction unaffected; *D*, dose (nM). (**A**) log–log dose–response relationships derived from the individual drugs (open circles), and the triple combination (closed triangles). Blue, TDF; red, FTC; orange; EVG; magenta, TDF-FTC-EVG. (**B**) Combination index (*CI*) plot comparing TDF-FTC-EVG (magenta open triangles) and TDF-FTC (teal open diamonds)*. CI* > 1 antagonism; *CI* = 1 (broken line), additive effect; *CI* < 1 synergism. (**C**) Dose-reduction index (DRI) plot for TDF-FTC-EVG. The DRI of 1 shown as a broken line represents no dose reduction relative to the drugs evaluated individually. Blue, TDF; red, FTC, orange; EVG.

**Figure 4:**
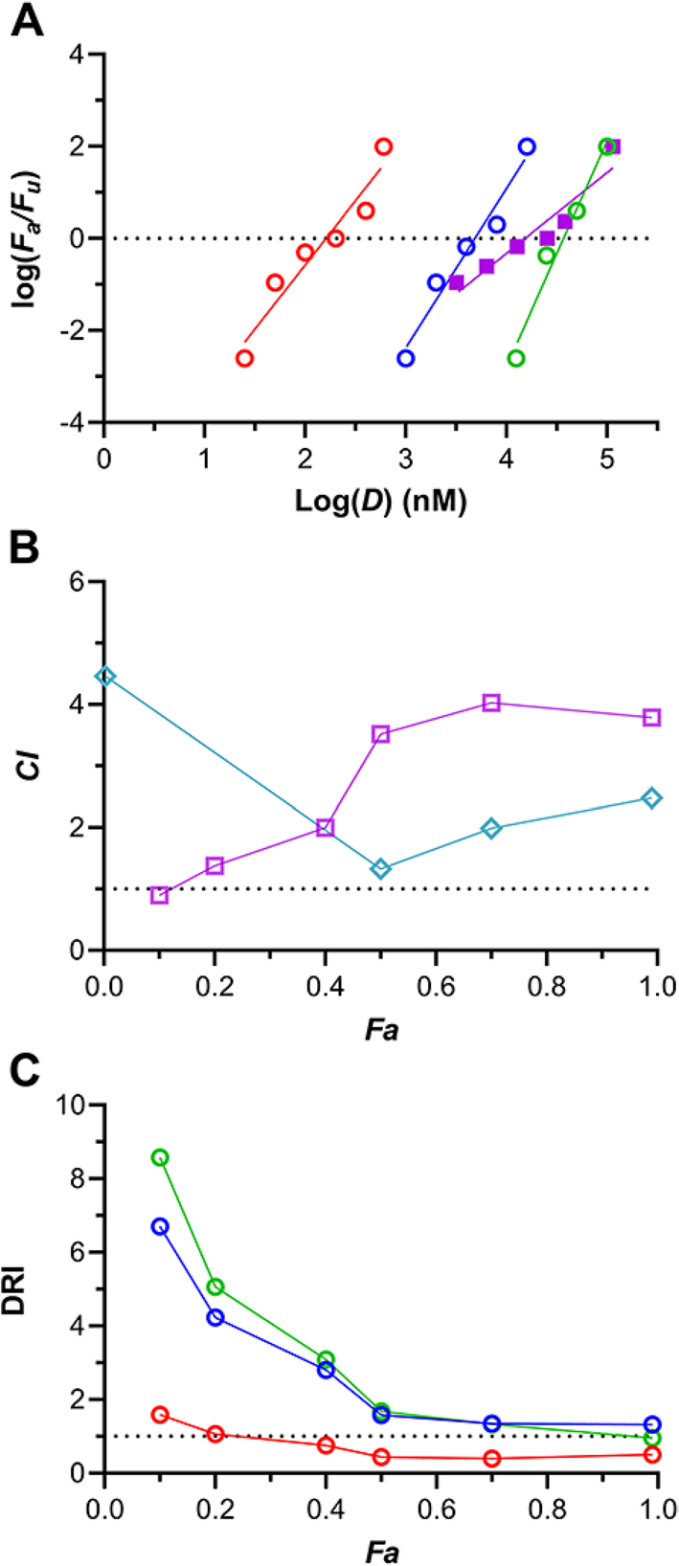
Median-effect model analysis of efficacy in vaginal HIV-1 prevention using BLT hu-mice (*N* = 10 per dosing group, 6 groups per study) and dosing with a triple drug combination consisting of TDF-FTC-C5A. *F*_*a*_, fraction affected; *F*_*u*_, fraction unaffected; *D*, dose (nM). (**A**) log–log dose–response relationships derived from the individual drugs (open circles), and the triple combination (closed squares). Blue, TDF; red, FTC; green; C5A; magenta, TDF-FTC-C5A. (**B**) Combination index (*CI*) plot comparing TDF-FTC-C5A (magenta open squares) and TDF-FTC (teal open diamonds)*. CI* > 1 antagonism; *CI* = 1 (broken line), additive effect; *CI* < 1 synergism. (**C**) Dose-reduction index (DRI) plot for TDF-FTC-C5A. The DRI of 1 shown as a broken line represents no dose reduction relative to the drugs evaluated individually. Blue, TDF; red, FTC, green; C5A.

The median-effect (Chou-Talalay) model based on mass action^21,22^ integrates foundational biochemical and biophysical equations (see “Discussion”). It does not require a preexisting knowledge of underlying mechanisms and is used for quantitative pharmacodynamic analysis of dose–effect relationships in complex biological systems. The analytical approach is particularly powerful for empirically unraveling the effects of two or more agents administered in combination. The median-effect model was applied to analyze data from vaginal HIV-1 prevention studies in BLT hu-mice dosed topically with TDF-FTC-EVG (Fig. 3) and TDF-FTC-C5A (Fig. 4). The goal of these studies was to determine if the slight-moderate antagonism observed previously^25^ between the nucleos(t)ide reverse transcriptase inhibitors (NRTIs) TDF and FTC (i.e., two drug combination) could be offset by adding a third drug with a different mode of action. The study premise was based on prior results where we have shown that addition of the bNAb VRC01-*N* to TDF-FTC in similar BLT hu-mouse studies led to a high synergistic effect (combination index, *CI*, tended to 0.4 as *F*_*a*_ approached 1.0) in preventing vaginal HIV-1 infection^27^.

The corresponding median-effect plots for the three-drug combinations follow the mass action principle (TDF-FTC-EVG, *R*^2^ = 0.904, Fig. 3A; TDF-FTC-C5A, *R*^2^ = 0.900, Fig. 4A). The quantitative assessment of drug combination effects was carried out using a combination index (*CI*) plot^21,22^. If the effect of the drug combination is simply additive, *CI* = 1. Antagonism is defined by *CI* > 1 and synergism by *CI* < 1. The combination indices at various *F*_*a*_ levels are presented below for TDF-FTC-EVG (Fig. 3B) and TDF-FTC-C5A (Fig. 4B), overlaid with the corresponding plots from the TDF-FTC dual combination. Unexpectedly, these results indicate that the third drug increased the antagonism relative to TDF-FTC, leading to a pronounced antagonistic effect—“strong antagonism” has been defined as 10 > *CI* > 3.3^22^.

The median-effect model was used to calculate the dose-reduction index (DRI) as a function of *F*_*a*_ (Figs. 3C, 4C) independently for all agents in the two combinations. The DRI represents a measure of the number of times the dose of each drug in the combination can be reduced (synergism) or needs to be increased (antagonism) at a given *F*_*a*_ compared with the doses of each drug alone^21,22^. A DRI > 1 is considered beneficial, but does not imply synergism because an additive effect or even slight antagonism also may also result in a DRI > 1. In both three-drug combinations, the DRI for FTC was less than unity for nearly all *F*_*a*_ values. However, for TDF the DRI was in the 1–2 range at high *F*_*a*_ values for both combinations. The highest DRI was observed for ETG (DRI = 2.2 as *F*_*a*_ approached 1.0, Fig. 3C), while the DRI for C5A seemed unaffected by the other drugs in the combination (DRI = 0.95 as *F*_*a*_ approached 1.0, Fig. 4C).

### Tenofovir disoproxil fumarate is a contributor to the antagonistic effect in three-drug combinations

**Figure 5:**
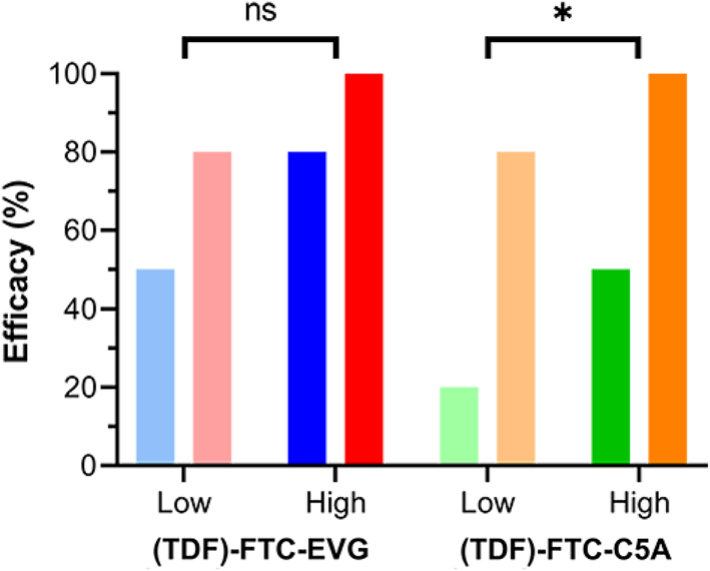
Vaginal HIV-1 prevention efficacy in BLT hu-mice increased when TDF was omitted. Blue, TDF-FTC-EVG; red, FTC-EVG; green, TDF-FTC-C5A; orange, FTC-C5A. Groups were compared using a one-sided paired *t*-test: ns, not significant (*P* = 0.10); *, significant (*P* = 0.035). Drug concentrations and efficacies are presented in Table 2.

Having established that severe antagonism existed between agents in both three-drug combinations tested here, we hypothesized that removing one of the two NRTIs would counteract this effect and lead to higher efficacy in HIV-1 prevention at a given drug concentration. Due to its lower potency (Table 1), we omitted TDF from the combinations, but kept the FTC-EVG and FTC-C5A concentrations the same as in the three-drug groups. We employed a low and high concentration spanning low (0.2–0.5) and high (0.5–0.8) effects in the previous three-agent studies (Table 2). In both regimens, the efficacies in the two-drug groups were higher than in the corresponding three-drug groups (Fig. 5, Table 2). A more dramatic effect (Δ*F*_*a*_, relative increase in efficacy for the two-drug regimen, Table 2) was observed for FTC-C5A (compared to FTC-EVG).

**Table 2:** Omission of TDF from the three-drug combinations increased the vaginal HIV-1 prevention efficacy in BLT hu-mice; Δ*F*_*a*_, relative increase in efficacy for the two-drug regimen.

[TDF] (µM)	[FTC] (µM)	[EVG] (µM)	[C5A] (µM)	*F*_*a*_ (%)	Δ*F*_*a*_ (%)
3067	367	67		50	
6900	825	150		80	
0	367	67		80	30
0	825	150		100	20
767	92		5500	20	
3067	367		22,000	50	
0	92		5500	80	60
0	367		22,000	100	50

We performed a one-sided paired *t*-test for matched pairs of each concentration with and without the use of TDF (Table 2). For the EVG group there was a trend toward increased efficacy (*P* = 0.10), while for the C5A group, the efficacy increased significantly (*P* = 0.035), as shown in Fig. 5.

## Discussion

The rational selection of drug combinations for vaginal (i.e., topical) HIV-1 PrEP needs to be based on empirical data that ideally maximizes synergism between the agents. By analogy, combination oral antiretroviral therapy (cART) for maintenance of viral suppression in individuals with HIV-1 infection typically involves regimens consisting of three agents. Orally administered TDF-FTC (Truvada, Gilead Sciences, Foster City, CA) represents the first FDA-approved regimen for HIV-1 PrEP^34^. We have shown that TDF-FTC infused vaginally can fully protect BLT hu-mice from vaginal HIV-1 infection^25^. We also demonstrated that long-acting delivery of TDF-FTC from IVRs achieved complete protection from simian/human immunodeficiency virus (SHIV) infection in the repeat low-dose vaginal exposure model using normally cycling female pigtailed macaques^35^.

It is not clear if multiple drugs are needed as part of an effective vaginal HIV-1 PrEP strategy, and whether such multi-drug regimens should be based on an NRTI backbone as in cART. The factors determining efficacy in preventing vaginal viral proliferation as part of PrEP may be different from those involved in HIV-1 suppressing in infected individuals as part of cART. Here, we have used rigorous, preclinical (i.e., in vivo) determination of the relative efficacy contributions from agents administered in combination to address this important question in an effort to select optimal regimens for further development as vaginal HIV-1 PrEP products.

The Chou-Talalay model, based on the median-effect principle of the mass action law, holds significant advantages over the traditional Kaplan–Meier design, as discussed previously^27^, and elegantly unifies four foundational biomedical equations, namely: Michaelis–Menten equation (enzyme kinetics); Hill equation (higher order ligand binding saturation); Henderson-Hasselbalch equation (pH ionization); and Scatchard equation (receptor binding)^21,22^. Application of the Chou-Talalay model to studying drug combinations does not require a priori knowledge of the mechanisms related to the biological effect, but can be used to quantitatively explore how the agents interact (i.e., addition, synergism, or antagonism). Using this approach we found that, when co-administered vaginally in BLT hu-mice, TDF and FTC displayed antagonism at high values of fractional inhibition (*CI* in the 2.0–2.5 range, when *F*_*a*_ ≥ 0.7; Figs. 3B and 4B)^25^. We also showed that adding a third drug using a different mode of action, the bNAb VRC01-*N*, resulted in pronounced synergism^27^. Consequently, we speculated that adding other agents with anti-HIV-1 mechanisms that could complement the NRTI foundation of TDF-FTC also would lead to synergistic effects when applied vaginally in BLT hu-mice. It therefore was surprising when we discovered strong antagonism when the ISTI EVG (Fig. 3) or the viral membrane-disrupting peptide C5A (Fig. 4) were added to the TDF-FTC combination. Omitting TDF, the less potent of the two NRTIs in preventing HIV-1 when applied vaginally in BLT hu-mice (Table 1), from the triple combination increased the HIV-1 prevention potency considerably (Fig. 5, Table 2). In the case of FTC-C5A, the increase was significant (*P* = 0.035). Future studies should determine the effect of omitting FTC, rather than TDF, from these combinations.

Efficacy outcomes in the BLT hu-mouse vaginal HIV-1 prevention studies presented here unexpectedly found that co-administering two drugs can be more effective than three, even if they are based on the established TDF-FTC backbone. These findings are counter-intuitive under the current paradigm of successful oral HIV-1 therapy using multiple ARV agents. It would be logical to expect established cART three-drug regimens for the suppression of HIV-1 in infected individuals to also be optimal for topical HIV-1 PrEP. Our results also suggest that for vaginal HIV-1 prevention, there may be additional factors governing the interaction of multiple drugs (i.e., promotion of synergism or antagonism) such as dispersal in the vagina, metabolism, and absorption, as well as the anatomic and pharmacologic compartments determining efficacy (e.g., cervicovaginal fluids versus immune cells in the vaginal mucosa). Combining EVG and FTC—both drugs inhibit different enzymes essential for HIV-1 replication in immune cells—was not as beneficial as combining C5A with FTC. In this case, the peptide C5A operates extracellularly by disrupting the viral envelope and potentially interfering with its ability to bind to host cells.

There are some limitations to our study. We have laid the foundation for further exploration here and in previous work^25–27^ by applying the Chou-Talalay model to quantitatively analyze the impact of drug combinations in vaginal HIV-1 PrEP. However, additional studies are needed before rational definition of drug regimens can be performed based solely on existing parameters such as potency, *m*-value, and mechanism. Our approach may not take into consideration potentially important pharmacokinetic parameters, such as drug or drug metabolite half-lives of elimination. For example, tenofovir diphosphate (TFV-DP) and emtricitabine triphosphate (FTC-TP) are the active intracellular anabolites against HIV-1 derived from TDF and FTC, respectively. In HIV-1 negative subjects (*N* = 19; 10 women) receiving daily oral Truvada (TDF, 300 mg; FTC, 200 mg) for 30 days, the terminal half-life of TFV-DP in peripheral blood mononuclear cells (PBMCs) during the washout phase was approximately double that for FTC-TP (4.4 days versus 2.3 days)^36^. These pharmacologic parameters, among others, could influence product performance in its intended method of use (e.g., period of “forgiveness” after the product is applied or removed) and were not necessarily captured in our studies. As with any animal model, physiologic and environmental (e.g., microbiome) differences between the mouse and human vaginal mucosa may limit the translational potential of our pharmacodynamic results. Finally, drug selection for vaginal HIV-1 prevention also will need to take the product’s characteristics into account. For example, on-demand vaginal dosing before and/or after vaginal sex (e.g., gels, fast-dissolving inserts, films) may benefit from different combinations of anti-HIV-1 agents, or even number of agents, compared with continuous dosing using long-acting regimens (e.g., IVRs).

Results from the current study illustrate how the complexities underlying the pharmacodynamics of drug combinations for vaginal HIV-1 PrEP can be unraveled using a simple, yet powerful mathematical model, while minimizing the number of animal used. The experimental and analytical approaches represent a translational platform for the rational, systematic selection of antiviral agents.

## Methods

### Materials

Tenofovir disoproxil fumarate (TDF), emtricitabine (FTC), and elvitegravir (EVG) kindly were provided by Gilead Sciences, Inc (Foster City, CA), or purchased from Selleck Chemicals LLC (Houston, TX). Maraviroc (MVC) was purchased from Selleck Chemicals LLC. The peptide C5A (purity ≥ 80%) was obtained from GenScript USA, Inc (Piscataway, NJ). All other reagents were obtained from Sigma-Aldrich (St. Louis, MO), unless otherwise noted.

### Animal care and ethics statement

The in vivo efficacy studies were carried out at the Department of Animal Resources (DAR), The Scripps Research Institute, using animal biosafety level 3 facilities, under protocols approved by the Institutional Animal Care and Use Committee at The Scripps Research Institute (Permit Number: 13-0001). The protocols strictly adhere with the recommendations in *the Guide for the Care and Use of Laboratory Animals of the National Institutes of Health*. Sodium pentobarbital anesthesia was employed in all surgeries, with a concerted effort to minimize suffering. Animal were sacrificed by cervical dislocation. The study is reported in accordance with Animal Research: Reporting of In Vivo Experiments (ARRIVE) guidelines.

### Generation of BLT hu-mice

BLT hu-mice were generated as described previously^25,28,37–39^. Briefly, human fetal liver and thymus tissues (*ca*. 1-mm^3^, Advanced Bioscience Resources, Alameda, CA) were implanted under the kidney capsule of female NSG mice (6- to 8-week-old, Jackson Laboratories, Ellsworth, ME). The mice were bred at The Scripps Research Institute, and each cohort was produced using tissues from a single donor. Magnetic bead selection (Miltenyi Biotec, San Diego, CA) was used to isolate CD34^+^ HSPCs from autologous fetal liver tissue. The purified CD34^+^ cells (Miltenyi Biotec, San Diego, CA) were phenotyped cytometrically^25,28,37–39^, and cryopreserved until injection (200,000–350,000 CD34^+^ cells) into mice 3 weeks after Thy/Liv implantation. Human reconstitution in peripheral blood was verified by flow cytometry using previously described methods^25,28,37–39^, and the degree of humanization was determined using a gating strategy described elsewhere^40^. Mice with an average > 65% of human CD45^+^ cells were selected to ensure successful HIV-1 infection.

### Vaginal exposure of BLT hu-Mice to HIV-1

Vaginal drug administration was carried out as described previously^25,27^ and summarized in Fig. 1. The employed drug concentrations in the single- and multiple drug dosing groups are provided in Supplementary Data S1–S4. Stocks of HIV-1 JR-CSF were prepared as previously described^28,37^ and standardized by p24 ELISA using the Alliance HIV-1 P24 ANTIGEN ELISA Kit (96 Test) (Perkin Elmer, Waltham, MA), according to the manufacturer’s instructions. Prior to inoculation, mice were anesthetized with isoflurane. Aliquots (5 μL) of drug solutions in PBS were applied vaginally through a pipet tip. The rear half of the mouse remained elevated during the procedure to reduce chance of back-flow from the vaginal cavity during the recovery. Within 30 min (typically 15–25 min) post-drug application, mice were vaginally challenged with HIV-1 (5 µL, corresponding to 200 ng of p24). This inoculum is a standard high viral load for successful vaginal infection (1 ng of p24 corresponds to ca. 10 infectious units). Methods used for the atraumatic vaginal HIV-1 challenge are described elsewhere^38,41–44^.

### Analysis of HIV-1 infection of BLT hu-mice

The infection status of BLT hu-mice was monitored by quantifying HIV RNA concentrations in peripheral blood (plasma) at weeks 1, 2, 3, 6 and 12 (Fig. 1) using one-step reverse transcriptase qPCR (Applied Biosystems custom TaqMan Assays-by-Design, ThermoFisher Scientific) according to the manufacturer’s instructions. Primers were 5-CATGTTTTCAGCATTATCAGAAGGA-3 and 5-TGCTTGATGTCCCCCCACT-3**,** and MGB-probe 5-FAM-CCACCCCACAAGATTTAAACACCATGCTAA-Q-3, where FAM is 6-carboxyfluorescein. Additional experimental details on the are presented elswhere^38,41–44^, and the assay sensitivity (limit of detection) was of 400 RNA copies per mL.

### Data analysis

Analytic simulations of dose–response curves using the median-effect principle and mass-action law, and its combination index theorem^21,22^ were carried out using CompuSyn^45^. *F*_*a*_ values of 0.0025 and 0.99 were used at 0 and 100% efficacy, respectively. Data were analyzed and plotted in GraphPad Prism (version 9.4.1, GraphPad Software, Inc., La Jolla, CA).

### Statistics and reproducibility

Power analyses were carried out using PASS 2021 (NCSS, LLC, Kaysville, UT). One-sided paired *t*-tests were conducted using RStudio (version 2022.07.1, RStudio, Boston, MA). For example, the paired *t*-test compared the difference in *F*_*a*_ for the same dose of two drugs (e.g., FTC-C5A), both with and without TDF (*N* = 2). A similar test (*N* = 2) was performed for FTC-EVG, both with and without TDF.

## Supplementary Information

Supplementary Information 1.

Supplementary Information 2.

Supplementary Information 3.

Supplementary Information 4.

Supplementary Information 5.

Supplementary Information 6.

## Data Availability

All data generated or analyzed during this study are included in this published article, and its Supplementary Information files.
