# Supplementary material for: Acute antagonism in three-drug combinations for vaginal HIV prevention in humanized mice
Source: Sci Rep. 2023 Mar 21;13:4594. doi: 10.1038/s41598-023-31695-5 (PMC10030891; doi:10.1038/s41598-023-31695-5)
Supplement: Supplementary file 1 — Supplementary Information 1. [file 41598_2023_31695_MOESM1_ESM.pdf]

Description of Additional Supplementary Files

File Name: Supplementary Data 1

Description: BLT hu-mouse MVC vaginal HIV-1 protection dataset (raw data, XLS)

File Name: Supplementary Data 2

Description: BLT hu-mouse EVG vaginal HIV-1 protection dataset (raw data, XLS)

File Name: Supplementary Data 3

Description: Round 1 BLT hu-mouse TDF-FTC-EVG and TDF-FTC-C5A vaginal HIV-1 protection datasets (raw data, XLS)

File Name: Supplementary Data 4

Description: Round 2 BLT hu-mouse TDF-FTC-EVG and TDF-FTC-C5A vaginal HIV-1 protection datasets as well as FTC-EVG and FTC-C5A vaginal HIV-1 protection datasets (raw data, XLS)

File Name: Supplementary Data 5

Description: Combination index (*CI*) and dose-reduction index (DRI) datasets underlying **Fig. 3** and **Fig. 4** (XLS)
